# Return-to-Work Experiences in Ontario Policing: Injured But Not Broken

**DOI:** 10.1007/s10926-023-10135-1

**Published:** 2023-09-21

**Authors:** D. Van Eerd, M. Le Pouésard, B. Yanar, E. Irvin, M. A. M. Gignac, A. Jetha, T. Morose, E. Tompa

**Affiliations:** 1 Institute for Work & Health, 400 University Ave, Toronto, ON M5S 1S9 Canada; 2 Public Services Health and Safety Association, 4950 Yonge St, North York, ON M2N 6K1 Canada; 3 Dalla Lana School of Public Health, University of Toronto, 155 College Street, Toronto, ON M5T 3M7 Canada

**Keywords:** Return to work, Work-related injury, Police, Qualitative research

## Abstract

**Purpose:**

Police officers and others working in police services are exposed to challenging and traumatic situations that can result in physical and/or psychological injuries requiring time off work. Safely returning to work post-injury is critical, yet little is known about current return-to-work (RTW) practices in police services. This study examines RTW practices and experiences in police services from the perspective of RTW personnel and workers with physical and/or psychological health conditions.

**Methods:**

We used a purposive sampling approach to recruit sworn and civilian members from several police services in Ontario, Canada. The recruited members had experienced RTW either as a person in a RTW support role or as a worker with a work-related injury/illness. We conducted and transcribed interviews for analysis and used qualitative research methods to identify themes in the data.

**Results:**

Five overarching themes emerged. Two pointed to the *context* and *culture* of police services and included matters related to RTW processes, injury/illness complexity, the hierarchical nature of police organizations, and a culture of stoicism and stigma. The remaining three themes pointed to the RTW processes of *accommodation, communication* and *trust-building*. They included issues related to recovery from injury/illness, meaningful accommodation, timely and clear communication, malingering and trust.

**Conclusions:**

Our findings point to potential areas for improving RTW practices in police services: greater flexibility, more clarity, stricter confidentiality and reduced stigma. More research is needed on RTW practices for managing psychological injuries to help inform policy and practice.

**Supplementary Information:**

The online version contains supplementary material available at 10.1007/s10926-023-10135-1.

## Background

Occupational injuries and illnesses, both physical and psychological, are common among police service members and may result in time off work [1–4]. Police officers have the highest percentage of days off work compared to other government workers, according to recent U.S. statistics [5]. In Ontario, Canada, police and firefighters have among the highest number of allowed workers’ compensation claims compared to other sectors [6]. While physical injuries remain a substantial burden for police services [6], mental health injuries and post-traumatic stress disorders (PTSDs) have gained attention as particular challenges in policing and other public safety organizations [7–12]. Martin [13] found the prevalence of PTSD was 8.0% in a sample of Canadian police officers, while a systematic review and meta-analysis determined the prevalence of PTSD among rescue workers worldwide to be 10.0% [14]. Carleton [7] found 23.2% of public safety personnel (PSP) screened positive for PTSD and had high rates of other mental health conditions, including mood, anxiety and substance use disorders. The numbers injured and the potential for severe and debilitating injuries and illness mean that recovery and return to work (RTW) in policing is an important organizational goal.

Some RTW studies that address mental health conditions suggest interventions should focus on the unique work environment of police services [15, 16]. Mumford [17] suggested a broad range of support services (e.g., alcohol abuse treatment, wellness training, mental health services) for police that are coordinated with the workplace, specifically regarding shift length and schedules. Arble [18] indicated police service members may have different coping behaviours than other PSP in PTSD recovery [18]. This may be due to the challenging physical demands and stress of police work, which pose barriers to RTW for police service members [15]. Plat [19], in a study examining PTSD treatment, recommended that successful RTW should be incorporated into treatment programs for police.

Earlier evidence syntheses about effective RTW following work-related physical and psychological conditions included few studies on the RTW of PSP [20–30]. Similarly, many empirical studies examining work absence and RTW among workers with psychological injuries and mental health conditions [26, 31–37] did not include PSP. While these syntheses and studies provide some evidence-based guidance on effective RTW programming in police services, their usefulness is limited by their lack of studies that included PSP. More recently, syntheses examining the effectiveness of RTW interventions for police and other PSP [38, 39] have been published. Edgelow [38] conducted an environmental scan of RTW programs for trauma-related conditions and found 13 programs relevant to trauma survivors, but found little information about the effectiveness of these programs. A narrative review of the literature on RTW programs after critical incidents [39] found a variety of different programs, but noted a lack of research on their effectiveness regarding RTW outcomes. The articles included in the narrative review tended to focus on stress and wellbeing and did not often include work outcomes.

Qualitative research has been suggested as an ideal method for studying RTW experiences [40–42]. A review of qualitative studies by MacEachen et al. [43] and subsequent qualitative studies [44–53] found that RTW is a complex process that requires trust, goodwill and clear communication. More recently, Edgelow [54] conducted a survey study of RTW experiences among PSP with a focus on psychological injuries. It found that PSP, on average, rated their workplace support as poor and reported this as a barrier to RTW, along with stigma and the difficulty navigating the RTW system. These barriers reflect the complexity of RTW and the need for clear communication and trust.

RTW programs in police services must address both physical and psychological injuries. Although RTW processes reported in the peer-reviewed literature are similar for physical and mental health conditions [55], it is not clear whether organizational practices and procedures that treat them similarly are considered optimal by returning police service members or those providing support in the RTW process.

Despite the growth of primary studies and evidence syntheses, a gap remains in the published literature on RTW in police services. In addition, little is known about current RTW practices in police services. To address this gap, this study used a qualitative thematic approach to examine current RTW practices and experiences of members with psychological and physical injuries in Ontario police services, from the perspective of both the workers and the RTW personnel supporting them. The knowledge drawn from the analysis gave rise to recommendations for improving RTW in police services.

## Methods

We used a qualitative research approach. We interviewed police service members who had experience with RTW as injured workers or support personnel. We thematically analyzed the interviews [56–58] to explore their experiences and gain an understanding of current RTW practices. Our approach was both deductive and inductive. This allowed us to consider organizational context, barriers and facilitators of the RTW process, and to provide recommendations of potential improvements drawn from the experiences of those involved in the process. We also included an interactive knowledge transfer and exchange component by involving a stakeholder advisory committee throughout the study.

### Stakeholder Advisory Committee

The stakeholder advisory committee included representatives from various police services and several police associations (Ontario Association of Chiefs of Police, Police Association of Ontario, Ontario Police Health and Safety Association). The committee helped with recruitment, reviewed the interview protocol to ensure we used correct terminology, and helped develop the messages arising from the study findings [59].

### Sample

We used a purposive sampling approach to ensure we recruited a broad representation of police service members working in Ontario: both sworn and civilian, across genders and age groups, with different types of injury (physical and/or psychological), and from police services of various sizes serving different-sized urban and rural communities (the latter to capture geographical/regional contextual differences). We included police service members who experienced work absences and RTW, as well as the personnel who supported them, such as supervisors/managers and RTW coordinators. This allowed us to capture a more complete set of RTW and practice experiences.

Our stakeholder advisory committee members aided in recruitment. Through their association networks, they reached out to potential study participants via emails, newsletters and e-blasts that included a link to an online recruitment survey. Interested participants completed the brief survey, providing their contact information and details about their current role in policing. Eligible participants were those who had experienced time off work due to an injury/illness or had a role in supporting the RTW of an injured member in their service. All participants gave their informed consent to participate. The study was approved by the University of Toronto Research Ethics Board (Protocol # 39059). Study methods and results reporting complied with the Consolidated Criteria for Reporting Qualitative Research (COREQ) Checklist [60].

### Data Collection—Interviews

An experienced qualitative interviewer (MLP) conducted semi-structured, 45–60-min telephone interviews. The interview questions were developed by the research team taking current scientific evidence on RTW into consideration. The stakeholder advisory committee then reviewed the questions and prompts to ensure we were using correct terminology that would make sense to the study participants. The questions asked participants about their experiences with work absences, RTW processes, RTW programs/practices and facilitators/barriers in the RTW process (see Appendix A). The questions were broad and open-ended, and the interviewer probed participants’ answers for details, taking cues from participants as to the most relevant and important issues to discuss.

### Analyses

After the interviews were transcribed, we (MLP, DVE, EI, MG, BY) conducted a thematic analysis using a codebook as per Braun and Clarke [56, 57] to identify and generate key themes related to RTW practices. We developed the codebook using the interview questions and prompts, as well as key findings from the peer-reviewed literature on RTW. We reviewed the interview data, analyzed it for content, and organized it into intermediary matrices [58]. We conducted a descriptive analysis of the qualitative data collected to understand participants’ views of RTW practices/programs, implementation processes, and perceived barriers and facilitators to implementation. We explored potential differences in experiences between those with psychological and physical conditions.

Through the thematic analysis [56, 57], we identified, generated and interpreted themes. We categorized the interview data into salient themes and used quotes to express participants’ experiences about RTW and implementation. We used NVivo to code the data and organize the text by themes (NVivo, released in March 2020 by QSR International Pty Ltd). The analytical team used reflexive methods, engaging in multiple discussions about the data and analysis. Our focus was to capture key insights relative to workplace RTW programs and practices from two perspectives: those of injured workers and those of RTW personnel. Our analysis and interpretation examined their core experiences, as well as their shared and divergent perspectives. In research team meetings, we discussed discrepancies in coding and interpretative differences and resolved them through discussion until we reached full consensus. We anonymized the analyzed content and presented illustrative examples to the stakeholder advisory committee for review and feedback on their relevance.

## Results

### Participant Characteristics

We conducted interviews with 49 participants from 13 Ontario police services. The services varied in size and population served and were located across different regions in the province. The purposive sampling approach allowed for a balance across sample characteristics and ensured breadth and depth in the interview data (see Table 1).

**Table 1:** Participant characteristics (n = 49)

Characteristic	Percent of sample (%)
Gender	
Male	53
Female	45
Age range	
18–34	12
35–44	35
45–54	37
55 and above	16
Role	
RTW personnel	41
Worker	59
Member type	
Sworn	61
Civilian	39
Worker injury type	
Physical	18
Psychological	33
Combination	49

### Overarching Themes

**Fig. 1:**
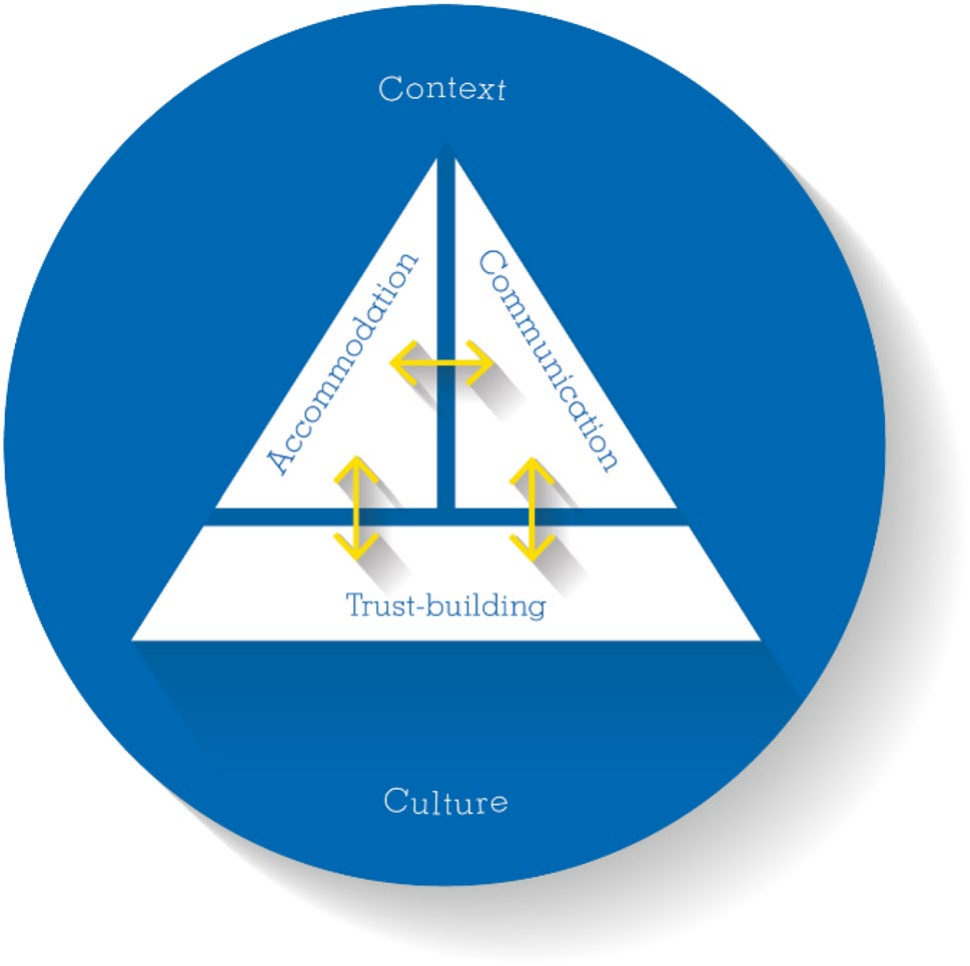
Representation of the themes generated about RTW experiences in policing

Our thematic analysis generated five inter-related themes about RTW practices in policing: context, culture, accommodation, communication and trust-building. Figure 1 depicts the overarching themes. The interrelated nature of the themes is shown in two ways: (i) the bi-directional arrows link the RTW process themes, which are depicted as pieces of a triangle; and (ii) a circle representing context and culture surrounds and shapes the other themes. The three RTW process themes: accommodation, communication, and trust-building were intertwined, each impacting the other themes, as well as being shaped by context and culture. The next sections describe the themes and subthemes, key aspects of their inter-relationships, and the similarities and differences in RTW practices for physical and psychological injuries. Representative quotes supporting the themes and subthemes are included in Table 2.

**Table 2:** Participant quotes supporting the overarching themes, organized by theme and subtheme

Theme	Subtheme	Quote
Context		“We understand that PTSD is a very difficult and tragic injury caused to individuals, especially police officers, but our position is [that] we as an organization are responsible to the community, to the taxpayer. We need regular updates on what the injury status is on that individual and what prognosis, if any, is in play… We need to make decisions… for public safety and the community.” (RTW personnel E005)
	*Complexity of injury*	“I’ve had a physical and a mental health [injury]. Unfortunately, the policies are all based around a physical injury. I found that being off on a physical injury, those policies made sense, but when I was off on a mental health-related injury, those policies really didn’t work that well. It felt like I was trying to fit a mental health issue into a physical issue. That’s where I feel my employer has fallen short. They really haven’t looked at return to work policies or being off on mental health issues… My time off on my psychological one was way harder just trying to deal with paperwork and all that stuff.” (Worker W025)
	*Complexity of injury*	“The process is very similar. The only thing that I do between the claims is physical injuries, typically, have a standard recovery timeline, you know, you break an arm it’s six weeks. You have different injuries, there’s a target date to when you would recover. With psychological claims there aren’t very many general recovery timelines, and one person may recover differently than another person, like the same injury. So, it’s kind of a work in progress. We leave a lot of room in between for modification and for altering things.” RTW personnel E007)
Culture		“You got to know very quickly what the hierarchy is. You have civilian females at the very bottom, male civilians above you, female officers, and male officers. That’s the hierarchy, and you are definitely treated differently, I’m not saying by every single person…30 years ago so it was a different mentality. But there are a lot of people who are just against, first of all, females in policing in any way, but then the civilians.” (Worker 011)
	*Stoicism*	“It’s funny, the week before I actually went off and sought the treatment and got sick, there was a senior officer […] I was in a meeting with him where he basically was slamming or talking negatively about people that go off for mental health reasons. And at that point I just thought, I was sick, I was like, ‘oh, there’s no way I can go off now.’ Because he was talking about people that we work with, people that we knew that were off getting help. So, that kind of stigma. And he’s a person that I genuinely like, I respect, I think the world of him. But in that case, he was very judgmental.” (Worker 004)
	*Stigma*	“In my role, I have lots of meetings with senior management. And in the past, before I went off work the first time… they have a whiteboard down at their end of the hallway in their offices with all the people who are off sick, on [injury leave] or other illnesses. And they call that board the broken toys. Those are the broken toy people. And they talk about what they’re going to do with them, how they’re going to get them back to count paper clips, and things like this. So, I’ve heard a lot of these comments many, many, many times over the years. So, when I went back, that’s what I felt like. That I was being looked at like I was a broken toy.” (Worker 013)
	*Stigma*	“Oh, there just is [stigma] with mental health, definitely. Hindsight is 20/20, but go off with a shoulder injury, and people are always like, hey, how is your shoulder? Go off with a mental health injury, and people don’t talk to you, or don’t check in because they don’t know what to say, or they … you know what I mean? There’s a complete difference between the two of them.” (Worker 022)
Accommodation	*Recovery from injury*	“The goal has got to be to get better. Number one, get better, number two, get back to work, I guess makes more sense. But I didn’t want to be off. And when I first talked to my doctor and he said to be off, I’m like, okay cool, so I’ll be back by summer. And he just laughed, no man, just relax. You’ll be back when you’re back. … Because I was doing therapy once or twice a week, I did everything. I maxed out my benefits. I did massage, I did hot yoga, I was doing acupuncture, I was trying everything and everything. Seeing a naturopath. Trying to get better, it was exhausting.” (Worker 004)
	*Recovery from injury*	“I was anxious to go back. It was me pushing to go back. I just wanted my life to be normal again. I missed work. I missed my co-workers. And, of course, you’re worried, out of sight, out of mind. Things are always changing at work. Policing is ever evolving. And you don’t really want to be out of the loop for too long because the longer you’re gone, the harder it is to get back because you’re already missing a lot of information and everything else. It was me who started that process.” (Worker 014)
	*Challenges of accommodation (availability)*	“We’re not a large Police Service…we try our best to put people in positions where they’re going to be most useful and get them back to work as soon as possible. But [we don’t] have countless desk positions. We have the units that we have for police officers, several units, but not to the level of some of the large Services that they can easily be brought back anywhere. We have to kind of figure that out each time what would be best based on their injury.” (RTW personnel E005)
	*Challenges of accommodation (meaningful work)*	“Workers’ comp, they wanted me to come back and start working four-hour shifts. The job I left before I was working 15-h shifts. Like four hours you basically put your boots on and you’re taking them off to go home. Then they’re like, oh, maybe you should do some gradual work in the office. No, I don’t want to do work in the office, that’s not my job.” (Worker 020)
Communication	*Genuine* *and* *timely* *communication*	“It was just an absolute mess. I just remember thinking, this is where I am as an employee, I’m a flowchart… It was like, are you kidding me, she couldn’t even have a conversation with me. It’s like as soon as you go off, they don’t even want to touch you. That’s how it felt.” (Worker 012)
	*Genuine* *and* *timely* *communication*	“We recently talked to [unit X] reminding them that those people shouldn’t be forgotten. We do have people that are off work with post-traumatic stress for lengthy periods. And sometimes it’s just a physical injury. And very, very often we hear back from those members that say, no one from my shift called me and my boss didn’t call me. No one’s calling me, you’re the only [person] that speaks to us. And there is no policy, but there should be policy about regular contact, I think.” (RTW personnel E010)
	*Clarity and consistency of process*	“Prior to my unit existing, one of the major complaints was that nobody from the service ever kept in touch with them, that they felt lost, there was no communication, and there was no check-ins. They just didn’t feel that they were appreciated when they were off work. They felt abandoned. For the people that I know of that are off work and I check in on, I don’t have a set timeline or a flag or anything, but some are every two weeks, some are once a month, some are longer than that, some are shorter than that depending on the situation and where they are in their recovery.” (RTW personnel E001)
Trust-building	*Lack of confidentiality*	“Because here, if you know anything about police services, nothing is secret, and everybody tells everybody everything, even though it’s private. There are privacy laws, blah, blah, blah, but everybody knows everything.” (Worker 012)
	*Lack of confidentiality*	“Sometimes you’ll hear chatter. Oh, yeah, they’re coming back for 22 days to get benefits and then they’ll be off again and that kind of thing. If I hear that, I would usually mention it and I would just say, listen, they’re coming back, whether they’re here 22 days or 23 days or six months, they’re coming back, so don’t be saying stuff like that to them and don’t be making this not a good place for them to be because that’s not good.” (RTW personnel E014)
	*Perceived malingering*	“My [coworkers] kept talking about how they kept seeing me in the gym, I looked good, again, [but] I’m just using the system. And I have to be in the gym strengthening muscles, so I didn’t have a choice, I had to go to the gym. And I hated to see my co-workers at the gym because I knew that’s the impression they had of me, so it was very difficult to try and put blinders on and focus on the job I had to do. It was very discouraging being off, especially my workplace, it really affected me. I wanted to go back so much but then all the stuff got back to me about how everyone kept thinking I was beating the system, taking advantage of the system, which isn’t the case.” (Worker 008)
	*Lack of trust*	"There’s also the fear of if you go, then you may not ever get promoted or get moved to another unit or another job. No one wants to throw that black mark on their file. […] They say they don’t hold it against you, but in more times than not, when you look at other people who get promoted and get moved around, the ones who went off or were suffering with something, never get moved. They never seem to move forward. So, there’s still something going on.” (Worker 010)

Participant RTW experiences varied considerably; some individuals reported a relatively uncomplicated RTW, while others described multiple challenges and setbacks throughout the process. The interview data reflected this variation, and the interrelated themes that emerged captured the range of experiences.

#### Context

Context played a key role in shaping RTW experiences. Successful RTW required not only input from various workplace parties, but often coordination with healthcare, insurance and/or compensation organizations outside of the workplace. Participants noted that policing is a service essential to public safety and felt that this had an impact on RTW practices.

##### Complexity of Injuries

Although both physical and psychological injuries could be complex, concerns about *complexity* and RTW were emphasized and discussed more often with respect to psychological injuries. Both workers and RTW personnel noted that RTW for psychological injuries was more challenging than for physical injuries, and they felt a uniform RTW approach for addressing both types of injuries was not suitable. Participants believed that current work disability practices were designed for, and more appropriate to, physical injuries and may not account for the complexity that comes with changes to mental health. When discussing the complexity of RTW for psychological injuries, participants often noted the need for more flexibility in timing and accommodation practices than was required for physical injuries.

#### Culture

When describing RTW experiences, many participants reflected on the culture within their organizations. They often remarked on the hierarchical nature of policing and referred to the “chain of command” as influencing RTW process and practices. At times, this hierarchy was thought to be influenced by other factors, including gender.

##### Stoicism

Participants often felt they needed to be *stoic* because the demanding nature of police work required strength and team focus. Many participants commented that policing left no room for weakness and having an injury was seen as a weakness. Related to strength was the concept of “having each other’s back,” particularly in potentially dangerous and life-threatening situations. Participants relayed concerns about how having an injury (particularly a psychological injury) as they navigated RTW would affect their ability to respond as needed to protect the public or other police members. Some members mentioned not discussing their injuries with anyone for fear of discovery, even with those in human resources (HR) or wellness units. This was compounded by a lack of confidentiality within their services, noted consistently by both worker and RTW personnel participants.

##### Stigma

*Stigma* was a common issue described by members, especially in relation to psychological injuries. Being perceived as weak was a key aspect of how stigma played out in the workplace, and weakness was often linked to injury as being “broken.” Participants from many different services used the term “broken toys” to describe how others perceived police members who were recovering from psychological injuries or had expressed mental health concerns. Injured workers also revealed that the stigma attached to injuries and recovery often delayed their decision to seek help or treatment and could make them reluctant to take time off. Perceptions of stigma also impacted the RTW process. Again, this was emphasized more for psychological than physical injuries.

#### Accommodation

Accommodation was described as a key element of successful RTW. Recovery from injury was considered important prior to workplace accommodation along with other practical challenges.

##### Recovery from Injury

Workers described the *recovery* process as requiring considerable effort and time. All participants remarked that recovery time and challenges were greater for psychological than physical injuries. Differing recovery times also caused communication challenges and were a concern because they affected when the accommodation process could begin. Many participants noted their considerable efforts to get better, although their efforts weren’t always recognized by others.

##### Challenges of Accommodation

Accommodation challenges stemmed from two key issues: (i) the availability of positions to accommodate the medical restrictions of injured workers, and (ii) the desire of injured workers to return to their original jobs (meaningful work). Regardless of the type of injury, workers described working hard to get back to their original role, get back to “normal,” return to their unit and partner, and resume the job that they were doing prior to injury. Workers described not wishing to appear “damaged,” a link to the stoic culture within policing. They also expressed concerns about jeopardizing their position or future career opportunities. In their experience, having an injury and being absent from work were cause for concern regarding career advancements and promotions. This was especially true for those with psychological injuries.

RTW personnel who were responsible for finding accommodated work commented on the challenges in finding positions that met workers’ medical restrictions. Medical restrictions varied but could include reduced hours, restricted contact with the public, or reduced exposure to specific stressors. Medical restrictions were considered a greater challenge for psychological than physical injuries. A key accommodation challenge involving psychological injuries was uncertainty about potential triggers, which could not always be accurately known or anticipated, even when the injured member felt ready to return to work. RTW support personnel from smaller services faced more challenges in finding accommodations because of the limited number of jobs/tasks available.

An additional challenge to finding accommodations was the desire of workers to return to their original jobs. This challenge often revolved around perceptions of meaningful work. Injured sworn members often considered their original position to be meaningful work and did not feel that “desk jobs” were meaningful, regardless of whether their restrictions ruled out other positions.

#### Communication

RTW was described as a complex process, requiring coordination with multiple workplace and non-workplace parties. This made clear communication vital to meeting accommodation needs and achieving RTW goals.

##### Genuine, Timely Communication

The need for *genuine* and *timely* communication throughout the RTW process was often expressed. Injured members spoke of communication that didn’t feel genuine, that felt more like a person going through a checklist of items to be discussed. RTW personnel spoke of the need to communicate with injured members frequently enough to ensure they had the information they needed at appropriate times, yet not so often as to overwhelm them, especially when they had a psychological injury.

##### Clarity and Consistency of Process

Another communication issue was related to the *clarity and consistency* of communication regarding the RTW process. Members reported being frustrated by a perceived lack of consistency in RTW practices over time or between themselves and others. RTW personnel acknowledged the need for consistency in RTW processes and communication, while also recognizing the importance of flexibility to deal with each RTW case individually.

When returning from a psychological injury, clear communication was mentioned as extremely important in helping injured workers understand the process. However, RTW personnel also recounted challenges related to establishing a schedule of contact and communication for those with psychological injuries.

#### Trust-Building

Issues of trust, lack of trust and the need to build trust were mentioned often as participants described RTW practices and experiences.

##### Lack of Confidentiality

A major concern among interviewees was the perceived *lack of confidentiality* within their services. Workers did not trust that details of their injury or recovery would be kept confidential. They commented directly about their lack of trust in HR for this reason, and many relayed stories of their injury status being disclosed across a unit or entire service without their consent. The concern about confidentiality was much greater for psychological than physical injuries.

Those in RTW roles commented that confidentiality was important, and they tried to maintain it in all cases. However, they also described situations when rumours and comments about injured members were made by work colleagues. When this occurred, RTW personnel often expressed concerns about how detrimental it was to injured workers and the RTW process.

##### Perceived Malingering

Another aspect of trust-building was *perceived malingering* or “milking the system.” Injured members reported hearing from others (e.g., their co-workers or supervisors) that they were milking the system and not truly injured. Often, when workers described their injury and RTW journey, they felt it was important to convey to the workplace and colleagues that they were not malingering but, rather, trying to recover and get back to work as quickly and safely as possible. These comments were related to *recovery* prior to the accommodation process, as well as workplace *culture*.

##### Lack of Trust

Workers also discussed a *lack of trust* in the people, departments or units commonly associated with the RTW process. Their description of distrust went beyond concerns about confidentiality and was linked with concerns about career advancement or movement within the service. Both workers and RTW personnel considered the lack of trust as a barrier to successful RTW.

## Discussion

This study is one of the first to explore RTW practices in police services in Ontario, Canada. Qualitative interviews were conducted with police service members, in a variety of roles, who had experience (positive or negative) with RTW, either as an injured worker or RTW personnel. A thematic analysis of the interviews revealed five overarching themes: context, culture, accommodation, communication and trust-building. These themes were inter-related and revealed current RTW experiences and challenges in policing.

The study’s findings are consistent with, and build on, previous qualitative findings about RTW. The themes of *context* and *culture* reflected the hierarchy within police organizations and the complex process of RTW [61]. Workplace culture has been identified as an important factor in RTW [45, 53, 61]. Policing has a unique culture relative to many other workplaces. A key finding within the culture/context themes showed that stigma, particularly injuries being seen as a weakness, could cause delays and was a barrier to the RTW process [45, 54, 62, 63].

Culture and stigma were also linked to differences in how physical and psychological injuries were perceived and managed. For example, those with psychological injuries were often referred to as being “broken” or being “a broken toy.” This concept was noted widely across various services. Referring to people as broken toys dehumanized members with injuries and minimized the seriousness of their experiences. It caused delays in seeking treatment and timely RTW. This study did not aim to examine stigma among police officers and other first responders or to find ways to change cultural perceptions. Therefore, we recommend more research be conducted to examine how to best address stigma in policing.

Within the theme of *accommodation,* we heard about the importance of recovery [64, 65]. Participants consistently noted that recovery from injury and successful RTW were important so that they could reliably do the job of protecting the public and their work colleagues. Also consistently noted were job-related and structural challenges in accommodating injured workers, which is a common problem in many sectors [50, 66–68]. Part of the challenge in policing related to sworn members not considering desk duties meaningful work; instead, they often pushed to go back to their original role. Recent research by Edgelow [54] also showed the PSP returning to work felt pressure to get back to their original role with little accommodation.

The theme of *communication* was considered a key element of the RTW process, as others have noted [46, 48, 50–52, 54, 69]. However, this project revealed that the concerns about communication between an injured member and the workplace further highlighted the difference between RTW for physical and psychological injuries. Both types of injury require timely contact, but additional challenges were noted for psychological injuries. Specifically, our findings revealed that consistent communication was often lacking for psychological injuries. Furthermore, a psychological injury may impact a worker’s ability to concentrate and process information and may be exacerbated when colleagues are concerned about their ability to work. The paperwork involved in RTW may also cause stress and may make it difficult to answer questions about current abilities and future work arrangements.

The theme of *trust-building* revealed issues related to confidentiality and the perception of malingering. Malingering has often been described in RTW studies [61, 70]. This study suggests a link between the organizational culture within police services and concerns about malingering. Participants noted that being labelled a malingerer undermined their efforts to recover from injury and RTW safely. While the concept of trust is often mentioned in qualitative research on RTW [45–47, 51, 61], the current study’s findings appear unique in pointing to the link between trust and job security/career advancement.

While current RTW practices for physical and psychological injuries are often similar, our study’s findings suggest they shouldn’t be. Police work is challenging and routinely exposes individuals to traumatic situations. Sutton and Polaschek [39], in a narrative review of the literature, recommended a number of programs (such as peer support, resilience and mindfulness training) that should be considered for RTW following a critical incident, while also noting that the evidence on the effectiveness for these programs was not strong. In addition, reintegration programs (peer-driven approaches to reduce long-term psychological injury) are a potentially promising RTW approach for psychological injuries [11]. A few Ontario police services noted in the interviews that they were exploring this approach but could provide few details as the programs were not yet implemented. Psychological injuries are not new in police services, but the increased emphasis on mental health in Ontario police services calls attention to the challenges of RTW [71]. Many police members in this study described experiences with both physical and psychological injuries. A key finding was that workers felt the RTW process was designed for physical injuries and did not work well for psychological injuries. Specifically, workers indicated their concerns about confidentiality were greater for psychological injuries and mental health issues, and this was often related to their fear about job security and promotions.

## Strengths and Limitations

This study has several strengths, key among them is that we gathered perspectives about RTW directly from those involved in the process: injured police service members/workers and RTW personnel. Conducting interviews with both injured workers in police services and those that support them provided a more comprehensive view of the RTW processes and challenges in policing. In addition, the study examined RTW for both physical and psychological injuries and compared how experiences and practices differed among them. The interviews yielded rich data about current RTW practices in Ontario police services and the experiences of members involved in RTW. Another strength was engaging with a stakeholder advisory committee, which aided in recruiting participants from police services of various sizes and differing locations, and also helped refine and disseminate the messages arising from the study’s findings.

A potential limitation of our study was the use of a convenience sample of police service members in Ontario. However, the purposive sampling approach helped to ensure our sample reflected a balance of injury types, service sizes and service locations. Future research needs to replicate the findings using samples in other jurisdictions, as well as conduct longitudinal research to follow RTW processes over time for workers with different types of injuries. In addition, although we had a balance of RTW experiences (from good to problematic), it is possible that those who volunteered to be interviewed may have different experiences related to RTW than those who did not. Specifically, people with more difficult or challenging experiences may have been more inclined to volunteer for the study and share their experiences than those whose RTW process unfolded smoothly. Finally, this study was conducted during the COVID-19 pandemic. This may have narrowed the number of potential individuals interested in participating and it certainly eliminated the possibility of conducting in-person interviews. Despite this, the study included rich descriptions of a variety of RTW experiences.

### Recommendations

The study’s findings suggest potential practice changes that could be implemented to improve the RTW process in police services, particularly for psychological injuries. This section describes recommendations as suggested by study participants, found in the literature by the research team, and supported by the stakeholder advisory committee. The recommendations are presented according to the overarching themes (see Fig. 1).

#### Context and Culture

Within police services, stigma was seen as a substantial barrier that affects the entire RTW process. Greater awareness of stigma (and related education) is needed at all levels of police services, particularly among those providing support to members with mental health conditions. Equating injury with weakness seems to be a key aspect of the stigma reported. Reducing stigmatizing language and building trust in police services was suggested by participants to improve the RTW process and timelines.

#### Accommodation

A key recommendation regarding accommodation is the need for more flexibility in the RTW process to allow for RTW plans customized to the individual, especially for psychological injuries. Accommodation challenges related to finding available positions for returning members requires an organizational approach that focuses RTW more on the worker (e.g., consistently including the injured worker in decision-making in a more transparent RTW process).

#### Communication

Clearly communicating with injured members about the RTW process is required. In addition, the frequency and mode of communication should be agreed upon with those who are off work. Improving communication about the RTW process could also help injured members better understand next steps and expectations. In addition, offering additional supports to members with psychological injuries was also recommended (e.g., having someone go through RTW forms with them).

#### Trust-Building

Building trust can be challenging, perhaps more so in hierarchical organizations like police services where the chain of command is an important operational element. Improving communication about the RTW process, along with strong efforts to reduce stigmatizing language assround injuries (especially psychological injuries), are important first steps. In addition, those involved in supporting RTW should be separate and independent from human resources. Police services can begin to address these concerns by increasing efforts to maintain confidentiality about injuries and RTW accommodations.

## Conclusions

This study reveals important findings about RTW in policing and suggests RTW practices in Ontario police services could be improved. While RTW research in other sectors has highlighted the importance of communication and trust in the accommodation process, the current study reveals unique aspects related to the police culture and concerns about processes for different injury types. Future research should focus on determining optimal communication approaches.

This research also highlights the need to examine the tension that exists between the mission of police to serve and protect and the objective of a safe return to work—and how to support both within police services. More research is required to better understand the link between trust and career opportunities in policing. Additional research using different methods, including larger longitudinal studies on workplace reintegration and RTW policies and practices, is necessary to examine key RTW elements and their effectiveness.

### Supplementary Information

Below is the link to the electronic supplementary material.

Supplementary file1 (DOC 105 KB)
