# Supplementary material for: Return-to-Work Experiences in Ontario Policing: Injured But Not Broken
Source: J Occup Rehabil. 2023 Sep 21;34(1):265–77. doi: 10.1007/s10926-023-10135-1 (PMC10899295; doi:10.1007/s10926-023-10135-1)
Supplement: Supplementary file 1 — Supplementary file1 (DOC 105 KB) [file 10926_2023_10135_MOESM1_ESM.doc]

**Appendix A – Interview protocol**

## a) Interview Guide for Employees

**Consent form procedures (verbal consent) to precede interview questions*

- - To begin, I would like to get a sense of your role and what your daily duties usually entail. Can you tell me a little bit about your job?
  - How long ago did you begin working at your current workplace/organization? When you first started working at your workplace, did you receive any training or orientation or document on RTW policies, practices, and procedures?
  - Have you had one or more absence(s)/leave(s) from work? If yes, how many absences/leaves have you had? Can you tell me a bit about the nature of the injuries (psychological or physical or a combination of both) and how long you were away?
  - Thinking about the time(s) you went off-work due to health reasons
    - How did you first report the injury or health reason that caused an absence?
    - Who did you turn to for help or assistance?
    - Were the steps you needed to follow clear to you?
    - Was there a policy or document that you used to guide this process? What was it? How did you find it? Did you find it or use it at the time it was most needed? Did you wish you had the material sooner?

1. Can you tell me more about your time off work and whether you had contact with the workplace [Potential probes: Did someone from your work stay in contact with you while you were off? Who? How often? Through which mode of communication?

- What was the content of the communication?
- How did this communication impact your recovery, if at all?
- Did you find it helpful? If so, how? If not, Why not?
- Was there a policy or document that outlined the expectations surrounding communication while off-work? Did you know about it or did someone give it to you? Tell me more.
  - Can you tell me more about what was involved in your decision to go back to work? What factors did you take into account?
    - Who did you turn to at the workplace, if anyone, for discussing this decision?
    - How was the support you received (or did not receive) important in your decision to return-to-work?
    - Is there anything else we should know about what you considered in your decision to RTW?
  - Before officially returning-to-work, did you have discussions with someone about supports, resources, services or accommodations to help you transition back to work? Can you tell me more?
    - Did you ask for supports, resources, services and/or accommodations, OR, did your [manager/supervisor/HR representative/association representative] provide you with different options of support, resources, services and/or accommodations? Why or why not? Can you tell me more?
    - Was there any policy or document used to guide the RTW process? What was it? Was it helpful? Why or why not?
    - Were there any specific procedures or practices that were followed in the RTW process? Can you tell me more about them? Were they helpful? Why or why not?
  - Now, can you think back to being back at work, after being off? What was that like?
    - *To be asked if supports were provided:* How did having that support impact your ability to do your work?
    - *To be asked if supports were not provided:* How did having limited or no support impact your ability to do your work?

**Conclusion:**

- - Overall, how was your experience with RTW at your workplace?
  - What was *most* helpful for you?
  - What was *least* helpful?
  - What advice, if any, would you give to supervisors or HR or disability managers to improve the RTW process and what would you tell them works or doesn’t work? [Potential probes related to who was/should be involved, content and amount of material given, timing of information]
  - Are there questions or details that we may have missed that are important to how you provide support with RTW?
  - Did I miss any questions you think I should ask to help us better understand your experiences?

## Interview Guide for Managers/Supervisors/HR Representatives/Association Representative

**Consent form procedures (verbal consent) to precede interview questions*

- - To begin, I would like to get a sense of your role and what your daily duties usually entail. Can you tell me a little bit about your job?
  - Please tell me about your experience in supporting workers RTW after an injury/sickness absence/leave, how much experience have you had with RTW?
    - Was the training you received enough to provide you with what you need to support others? Please explain. If no, what [additional] kind of training or policies and practices do you think are needed to support others?
  - Does your workplace have a RTW process or program?
    - If yes, when did first learn about this process/program? Was it part of your training or did you learn about it after an injury occurred in the workplace or at some other time? Are there any changes you would recommend in how and when people learn about the RTW process/program?
    - If yes, can you tell me more about how it works?
    - If no process/program, why isn’t there one? How is RTW carried out at your workplace? Can you give me examples of how it has unfolded in the absence of a process or program? Are there changes you think should be made – if so, what?
  - When an employee leaves work, do you stay in contact with them?
    - If yes, how often? What is the mode of communication?
    - If yes, what is the purpose of the communication? What do you typically discuss with the employee? Does it make a difference? If so how? If not, why not?
    - Does the process differ based on the nature of the injury? Why/why not?
    - Is there a policy or document that outlines how this communication should take place? Is the information all in one place? Where can it be found? Are there separate policies or documents for physical and psychological injuries?
  - Before the employee officially RTW, did you have discussions about supports, resources, services or accommodations to help them transition back to work? Can you describe this process and give some examples?
    - If yes, how does this discussion take place?
    - If yes, who is involved in this discussion?
    - If no, why not?
    - Does the process differ based on nature of injury? Why/why not?
- What is the process, if any, for deciding which supports, resources, services and/or accommodations are provided? Can you give me examples?
  - - Does the process differ based on nature of injury?
    - Is there a policy or document that outlines the steps to this process? Is the information needed all in one document? Where can it be found?
    - Are employees involved in this decision?
      - If yes, how? Does this work? Is their involvement the same or different depending on the nature of injury?
      - If no, why not? Does this work?
- Do you follow any procedures moving forward with providing your employee with supports, resources, services and/or accommodations?
  - - - To be asked if yes: What are they? Do the procedures differ depending on the nature of injury? Why/why not?
      - If there are no supports, resources, services and/or accommodation offered, why not?
- How do you address the RTW of an employee with coworkers? Can you give examples? Why is this approach used? Does it help? Is the same approach used regardless of the nature of the injury?
  - - What do you communicate to coworkers regarding the RTW of an employee? Does it help? Does this differ based on the nature of injury?
    - Is there a policy or document that informs this communication? Is the information all in one place? Where can it be found? Are there separate policies or documents for physical and psychological injuries?
- Once the employee returns, do you have any programs in place to help them stay- at-work? Can you give examples/tell me more? Are the programs the same regardless of the nature of injury? Are they helpful? Would you recommend changes?

**Conclusion:**

- Is the RTW process (or program) at your workplace effective at getting employees back to work? If so, how? If not, explain why.
- In your experience, what supports, resources, services and/or accommodations have been the most beneficial to your employees?
- Based on your experience, what are the key facilitators for implementing a return-to-work process (or program) at your workplace? [prompt if needed: supports, resources, services]
- Based on your experience, what are the key barriers to implementing a return- to-work process (or program) at your workplace?
  - How can these barriers be best addressed?
  - Which tools would help facilitate RTW in your workplace?
- Are there questions or details that we may have missed that are important to how you provide support with RTW?
- Did I miss any questions you think I should ask to help us better understand your experiences?
